# Prevalence and correlates of partner violence among adolescent girls and young women: Evidence from baseline data of a cluster randomised trial in Tanzania

**DOI:** 10.1371/journal.pone.0222950

**Published:** 2019-10-08

**Authors:** Daniel Nyato, Jacqueline Materu, Evodius Kuringe, Jeremie Zoungrana, Deusdedit Mjungu, Ruth Lemwayi, Esther Majani, Baltazar Mtenga, Soori Nnko, Grace Munisi, Amani Shao, Mwita Wambura, John Changalucha, Mary Drake, Albert Komba

**Affiliations:** 1 Department of Sexual and Reproductive Health, National Institute for Medical Research, Mwanza Centre, Tanzania; 2 Jhpiego Tanzania - An Affiliate of Johns Hopkins University, Dar-es-Salaam, Tanzania; Washington University in St. Louis, UNITED STATES

## Abstract

**Background:**

Little has been documented about partner violence among adolescent girls and young women (AGYW) who are out of school, a factor associated with HIV acquisition. To understand areas for prioritising HIV prevention intervention efforts, we explored the prevalence and correlates of partner violence among out of school AGYW in Shinyanga, Tanzania.

**Methods:**

A cross-sectional analysis of data from AGYW aged 15–23 years recruited in a cluster randomised trial conducted between October and December 2017 was used to examine correlates of partner violence. Data were collected through an Audio Computer-Assisted Self-interview. Multivariate logistic regression analysis was used to evaluate the association.

**Results:**

2276 (75.5%) AGYW were sexually active. Of these, 816 (35.9%) reported having experienced violence from partners in the last six months. After adjusting for other covariates, being formerly married (AOR = 1.55, 95% CI:1.02, 2.37), having children (AOR = 1.79, 95% CI:1.47, 2.16), anxiety and depression symptoms (AOR = 3.27, 95%CI: 2.15, 4.96), having engaged in sex work in the past six months (AOR = 1.92, 95% CI: 1.45, 2.53) and economic deprivation (AOR = 1.61, 95% CI: 1.34,1.92) were significantly associated with partner violence.

**Conclusions:**

Almost one in three sexually active AGYW had experienced partner violence in the 6 months preceding the survey. The findings underscore the need for future research to focus on understanding the reasons and dynamics underlying high level of partner violence among AGYW. Furthermore, there is a need for implementing intervention programs that aim to reduce economic deprivation among AGYWs and address social norms and structures perpetuating violence against AGYW.

**Trial registration:**

**ClinicalTrials.gov—ID** NCT03597243.

## Background

Partner violence against women is a major global public health problem and a human rights violation [1–4]. The World Health Organisation (WHO) defines partner violence as “the range of sexually, psychologically and physically coercive acts used against adult and adolescent women by current or former male partners” [5]. Global estimates show that almost a third of women aged 15 and above will experience physical and/or sexual violence over their lifetime [3]. Sub-Saharan Africa (SSA) is among regions with the highest rates of lifetime physical and/or sexual violence (36%) [6]. In Tanzania, research has documented unacceptably high levels of physical and sexual violence by partners [7, 8]. Data from the Demographic and Health Survey and Malaria Indicator Survey (DHSMIS) 2015–2016, shows that 44% of women aged 15–49 years have ever experienced physical and/or sexual violence at some point in their lives [9]. About 56% of ever-partnered women in Mbeya city and 41% in Dar es Salaam had ever experienced physical or sexual violence at the hands of a partner [7]. In addition, unmarried girls and young women equally experience partner violence in Tanzania, with DHSMIS data showing 16% of never-married women to have ever experienced physical violence, and 9% to have ever experienced sexual violence perpetrated by their partners [9].

Gender inequalities, rooted within cultural norms and beliefs on gender roles and power relations, have a profound influence on experiences of partner violence [10]. In Tanzania, partner violence is so commonplace that in some places it is accepted as justifiable even by women themselves [11]. For example, in a qualitative study involving young men and women aged 16–24 years in Dar es Salaam, an ideal woman was described as one who is loyal to her partner, sexually submissive and home-bound [12]. Such norms are internalised in ways that it is not uncommon for both men and women in Tanzania to condone partner violence as a normal part of intimacy [13–15]. In addition, young women who enter early sexual relationships can have higher rates of partner violence compared to older women, as seen in a community-based study in Uganda in which women who become sexually active younger than 15 years faced almost twice the risks of violence compared with those who became sexually active at or after 18 years [16].

Health impact of women’s exposures to partner violence are multiple [17]. Studies show that partner violence is associated with mental health problems including depression, anxiety, phobia, post-traumatic stress disorders, drug abuse and alcoholism [18], and is a leading cause of homicide deaths in women [19]. Recent evidence suggests that partner violence increases HIV risk through various mechanisms [17, 20, 21]. Forced sexual intercourse with an HIV-positive male partner can lead to direct transmission of HIV infection. The risk of HIV transmission increases with physical trauma, vaginal lacerations and abrasions [20]. HIV transmission is likely to be higher among adolescent girls and young women during sexual violence because girls’ vaginal tracts are immature and tear easily during sexual intercourse and more so during a forced penetration [22]. Trauma associated with violent experiences can also impact negatively on later sexual behaviour by increasing the women’s HIV risk-taking behaviour, which increases susceptibility to HIV infection [23–25]. For instance, experiences of violence can lead to having unprotected sexual intercourse out of fear that asking for condom use could lead to violence or substance use as a coping mechanism for trauma[26]. The evidence further shows that partner violence undermines antiretroviral treatment uptake and adherence [21].

There is an emerging body of research suggesting that interventions that directly aim to challenge gender inequalities and power imbalances help to lessen partner violence and HIV risk behaviours. These include empowering women [27], working with men to challenge inequitable gender norms [28–30], and engaging men and women in discussions about gender inequality and its implications [31]. While the evidence is promising, less is known about the needs and applications for HIV prevention associated with partner violence among adolescent girls and young women.

The current study adds to the body of knowledge on the prevalence and correlates of partner violence among a group of out-of-school AGYW in Tanzania. The literature addressing this particular group is limited. The findings will be useful to program planners and policymakers seeking to tailor HIV prevention approaches to be most appropriate to the needs of this vulnerable population.

## Methods

### Study setting

The government of Tanzania through the Ministry of Health, Community Development, Gender, Elderly and Children (MoHCDGEC) is championing and coordinating efforts to address HIV prevention and violence against women. The Sauti project, a comprehensive community outreach program, has been implemented by the MoHCDGEC and Jhpiego in 14 regions of Tanzania since 2015. The program aims to reduce vulnerabilities to HIV infections among AGYW and other key and vulnerable populations (KVP) by providing biomedical, behavioural and structural interventions tailored to address the specific needs of KVP. Biomedical interventions include the provision of HIV testing, STI screening, family planning (FP) services, screening for TB, gender-based violence (GBV) and alcohol and drug abuse at community-based delivery points. People found to be HIV positive are linked to care and treatment services.

Peer educators deliver behavioural interventions provided to AGYW through Sauti. Sauti regularly trains peer educators and conduct weekly meetings to offer life skills, HIV and FP-related counselling messages, and to provide a forum for savings and loan groups. An unconditional cash transfer program for AGYW is conducted in Shinyanga region; in which AGYW receive the equivalent of about 33 USD per quarter. In addition, Sauti uses campaigns and community forums to foster community discussions about gender-based violence and to reinforce positive gender norms as related to HIV-risk behaviour.

Sauti project uses the Start Awareness Support Action (SASA!) strategy to address partner violence in Shinyanga region. SASA! stresses challenging gender norms and power imbalances through community interventions (creation of male champions against partner violence, key messages and discussion provided in community forums, drama, film), and individual level interventions (peer counselling and support, screening for violence, individual counselling). Those found to be experiencing partner violence are linked to referral service points for services including trauma management, emergency contraceptives, and post-exposure HIV prophylaxis and police intervention. Sauti also works with community leaders, health care providers and the police force to improve the care afforded to women who experience violence.

### Parent study—The CARE study

A study–“Cash Transfer to Adolescent Girls and Young Women to Reduce Sexual Risk Behaviour–an Impact Evaluation (CARE)—is being conducted to understand how cash transfer and other interventions synergistically mediate to reduce the risky sexual behaviour in Shinyanga, Tanzania.

This paper is based on a cross-sectional analysis of baseline data from the CARE study. The analysis aims to answer the following two-pronged research questions: What is the prevalence of partner violence among sexually active AGYW enrolled in a cluster randomised trial (CARE study) in Shinyanga region, Tanzania? And what are the risk factors associated with partner violence among this population?

#### CARE study design

CARE study is a two-arm cluster randomised trial involving 30 villages (fifteen intervention and fifteen control) in Kahama municipal council, Msalala and Ushetu district councils in Shinyanga region, Tanzania. The villages in each district were eligible for CARE study if they were Sauti project villages with at least 110 AGYW who are out of school, and AGYW in that community were potential cash transfer recipients. A village was the unit for a cluster. Thirty matched villages were randomised to either receive cash transfer delivered over 18 months in addition to other HIV interventions (n = 15) or other HIV interventions without cash transfer (n = 15). High HIV risk was decided based on the presence of mines, fishing areas and plantations. During baseline study, participants in all clusters were receiving biomedical (i.e., HIV testing and counselling), behavioural (i.e., social and behaviour change communication (SBCC) and structural (i.e., financial literacy, micro-business development skills and community banking) services as part of Sauti project. Cash transfer was not being provided yet.

### Study population and sample size

Participants were included in the current study if they were: 15–23 years old, out of school (either never been to school or dropped out at least one month before study enrolment), and had graduated from ten hours of SBCC curriculum sessions. Young women (15–24) are of interest to this study because they have been found to be twice as likely to acquire HIV as young men of the same age group [32, 33].

Potential participants were approached during SBCC sessions in their training groups to be informed about the study, its related procedures and eligibility criteria for participation. After the 10 hours of SBCC, the potential participants approached the study registration desk for pre-screening consent and screening.

Three thousand one hundred and five (3105) potential participants presented at the registration desk to be screened for eligibility. Among them, 3071 potential participants met the inclusion criteria. Of these, 3055 participants consented to take part in the study. Of those who consented, 3014 participants completed the interviews using ACASI.

### Study procedures

During the baseline survey, consenting/assenting AGYW completed a structured interview in the form of Audio Computer-Assisted Self-interview (ACASI)[34]. Questionnaire in English was translated to Kiswahili and back-translated to assess the quality of the translation. Each question in Kiswahili was voice recorded by a female research assistant, and later all voice recorded questions, and text questions were programmed into tablets (S1 Questions in Kiswahili).

Trained female interviewers showed participants how to navigate on ACASI questionnaire on the tablet. Participants were allowed to practice answering questions until they felt comfortable starting the interview. Interviews were conducted in a private location and help was provided by trained female interviewers, whenever needed. For accuracy and reliability of responses, two test questions were embedded within the questionnaire. In particular, we asked the following questions: Are you in Tanzania now? Are you a boy or a girl? Participants who wrongly answered both questions were excluded from the analysis. The questionnaire included information about demographics, a risk affinity score, sexual risk behaviours, income and intimate partner violence. Following the completion of the interview, participants were compensated 10,000/- Tanzanian shillings (equivalent USD 4) as a refund for transport expenses and time spent.

### Measures

#### Socio-demographic questions

Participants were asked to report their age, sex, education, marital status and parity.

#### Economic deprivation

Participants were considered economically deprived if they met at least one of the following criteria: stayed a day or gone to bed hungry (i.e. without having dinner) due to lack of food within the last four weeks; or if they were living in a household supported by a social welfare program of Tanzania Social Action Fund (TASAF). TASAF funds households identified as living below the poverty line, which is equivalent to 1 US dollar per day.

#### Sex work

Participants were categorised as sex workers if they had negotiated for money in exchange for sexual intercourse in the last six months and if the income accrued through sex comprised of 50% or more of their overall income.

#### Mental health

Depression symptoms were measured using a screening tool developed and validated by Kroenke and colleagues [35] called the Patient Health Questionnaire (PHQ-4). The PHQ-4 tool records symptoms of anxiety and depression on a four-item scale (2 for anxiety and 2 for depression) rated on a 4-point Likert-type scale. The PHQ-4 tool was incorporated in the ACASI questionnaire and was graded as normal (0–2), mild (3–5), moderate (6–8), and severe (9–12) [35]. The PHQ-4 tool has not been validated in Tanzania; however, the internal consistency of PHQ-4 was checked through Cronbach’s alpha using polychoric correlation matrix and obtained a value of 0.77.

#### Partner violence

The questions on partner violence were limited only to those who reported having had vaginal or anal sex in the last six months. Partner violence was assessed using an adaptation of the World Health Organisation violence against women instrument [4], which provides clear measures of each typology of violence (physical, emotional and sexual) as shown in Table 1. In this study, AGYW who experienced at least one form of violence (emotional, sexual or physical) from a partner were defined as experienced partner violence for the last six months. Those who did not experience any form of violence were defined as never experienced partner violence over the previous six months.

**Table 1:** Questions used in the CARE study to document partner violence.

	Questions
**Emotional violence**	**In the last six months, has any of your partner ever said or done anything to**
	**1. Humiliate you in front of others?**
	**2. Threaten to hurt you or someone you care about?**
	**3. Insult you or make you feel bad about yourself?**
**Physical violence**	**In the last six months, has any of your partner ever used his hands /an object to**:
	**1. Hurt you physically? Like**:
	**a. Pushing you**
	**b. Shook or threw something at you**
	**c. Slap you**
	**d. Punch you with a fist or with something that could hurt you**
	**e. Kick**
	**f. Drag or beat you up**
	**g. Choke you or burn you on purpose**
	**h. Threaten to attack you with a knife, gun or weapon**
**Sexual violence**	**In the last six months, were you ever**:
	**1. Forced to have sex against your will?**
	**2. Had sex even when you did not want because of fear of what your partner would do?**
	**3. Forced to do something sexually that you felt degrading or humiliating**

#### HIV testing

HTC was conducted on-site following Tanzanian HIV testing and counselling guidelines [16] and following NIMR standard operating procedures. HIV testing was performed using SD Bioline HIV-1/2 3.0 (Standard Diagnostics, Inc., Korea) and UniGold Recombigen HIV test [Trinity Biotech, Bray, County Wicklow, Ireland] rapid tests. Discordant results were confirmed using BioElisa HIV 1+2 Ag/Ab Test (BiokitS.A, Barcelona, Spain) at NIMR, Mwanza centre laboratory.

### Data analysis

Data were managed and analysed using R 3.4.3 and SAS 9.4 (SAS Institute Inc.; Cary, North Carolina). The primary outcome variable was partner violence: association with HIV-positive status, socio-demographic characteristics and sex work were explored. Descriptive analysis was conducted to describe the socio-demographic characteristics, the prevalence of HIV, sex work and different types of partner violence. Logistic regression was conducted to assess the association between socio-demographic characteristics, sexual risk behaviour and HIV prevalence on partner violence, controlling for possible confounders. Variables in the univariate analysis that showed a significant effect on the dependent variable were included in the multivariable analysis. Unadjusted and Adjusted Odds ratio (AOR) with 95% confidence interval (95% CI) were computed and reported where appropriate. As violent behaviours may not be independent within villages, thus a robust estimation of variances was used to account for correlation[36, 37]. Statistical significance was considered at a p-value less than 0.05.

### Ethical considerations

CARE study was granted ethics approval by the Medical Research Coordinating Committee (MRCC) of the National Institute for Medical Research (NIMR) in Tanzania (NIMR/HQ/R.8c/Vol.II/841), and Institutional Review Board of the Johns Hopkins University (IRB00007976). Approval to work in the study communities was obtained through official permission from respective local government offices and leaders after authorisation from the regional and district government authorities. All participants had to provide written informed consent for participation in the study for those who were at least 18 years old, while those below 18 years, written assent was obtained from participants and consent provided by their parents or guardians. Married young women below 18 years were considered as emancipated minors and therefore consented for themselves. Training of field staff included human subjects’ protection, the unique vulnerabilities of AGYW and safety procedures. Sensitisation for data collection was conducted in close collaboration with local community-based organisations and local government representatives to increase acceptability and safety.

## Results

### Description of study participants

The trial interviewed 3,014 AGYW at baseline. However, one participant was excluded from data analysis due to failure in discernment questions. Hence a total of 3013 AGYW were included at baseline. Of the participants, 75.5% reported having vaginal or anal sex and these were the participants described in the analysis. The mean age of AGYW was 20 years (standard deviation of 2.3 years), with 65.1% aged 20 to 23 years. Half of the study participants had a primary school education (51.4%), more than a half were married (60.0%), and had children (71.6%). All AGYW were tested for HIV, and 4.0% were HIV positive (Table 2).

**Table 2:** Demographic characteristics of study participants.

Factor	Total (N = 2276)
	n (%)
**Age category**:	
15–19	795 (34.9)
20–23	1481 (65.1)
**Marital status**:	
Single	742 (32.6)
Currently-married	1365 (60.0)
Formerly-married	169 (7.4)
**Education status**:	**674 (29.6)**
Never been to school/Incomplete primary school	1170 (51.4)
Completed primary school	432 (19.0)
Completed/In completed secondary school	
**Having children**:	**647 (28.4)**
No	1629 (71.6)
Yes	
**HIV status**^a^:	**2175 (96.0)**
Negative	91 (4.0%)
Positive	
**Partner violence**:	**1460 (64.2)**
No	816 (35.9)
Yes	
**Emotional violence**:	
No	1596 (70.1)
Yes	680 (29.9)
**Physical violence**:	
No	1878 (82.5)
Yes	398 (17.5)
**Sexual violence**:	
No	2157 (94.8)
Yes	119 (5.2)
**Anxiety-depression symptoms**^b^:	
Normal	868 (38.1)
Mild	759 (33.4)
Moderate	491 (21.5)
Severe	158 (6.9)
**Sex work in the last six months**^c^:	
No	1889 (83.0)
Yes	387 (17.0)
**Economic deprivation**^d^:	
No	1536 (67.5)
Yes	740 (32.5)

^**a**^10 observations missed (AGYW they left field site before HIV testing);

^**b**^Combination of anxiety and depression;

^**c**^Paid some money in exchange for offering sexual intercourse;

^**d**^Went to bed hungry due to lack of food for the past four weeks or received TASAF support

### Prevalence of partner violence

Among sexually active participants, 35.9% reported experiencing any form of partner violence in the last six months. The most common form of partner violence among sexually active participants was humiliation/emotional violence (29.9%), followed by physical abuse/violence (17.5%), and then rape or sexual abuse (5.2%) (Table 2).

**Fig 1:**
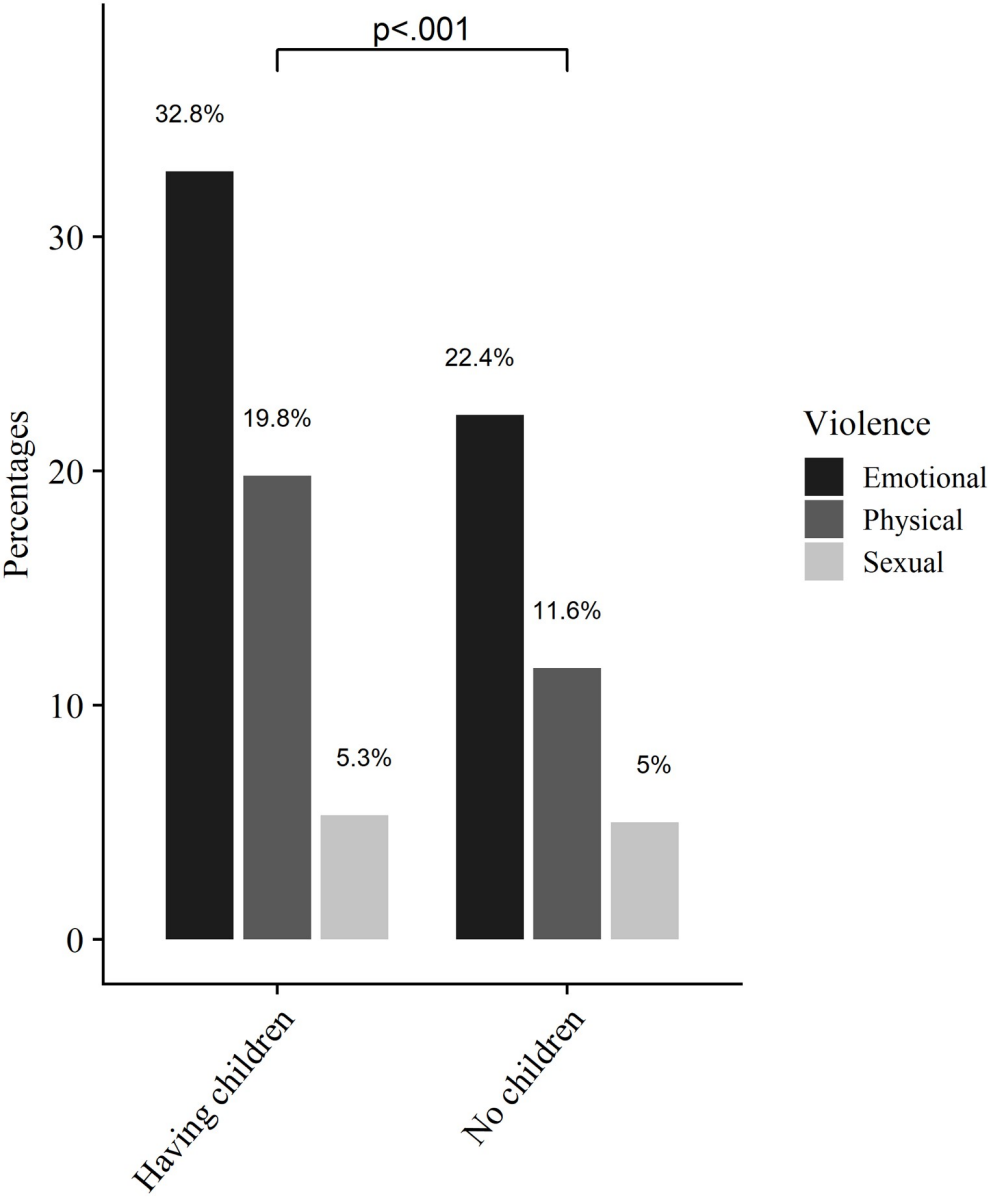
Sex work by violence among AGYW.

Of the AGYW who were formerly married and those who had children, 52.1% and 39.4%, respectively, reported experiencing violence from their partners in the past six months. Among participants who screened positive for severe and moderate anxiety and depression (PHQ-4) symptoms, 53.5% and 47.3% respectively, experienced violence from their partners in the last six months. Among HIV positive AGYW, 42.9% reported experiencing partner violence. Of those reporting economic deprivation, 45.5% reported having experienced violence from their partners. In addition, among those who reported negotiating for money before they had sex, 49.4% reported having experienced partner violence (Table 3, Fig 1). Among AGYW who reported having engaged in sex work and experienced violence from their partners, 44% experienced emotional, 26% physical and 12% sexual violence (Fig 1).

**Table 3:** Association between partner violence and their correlates (n = 2276).

	n(%) reporting partner violence	Unadjusted OR (95% CI)	Adjusted OR (95% CI)
**Age (Years)**:			
**15–19**	**247 (31.1)**	**1**	**1**
**20–23**	**569 (38.4)**	1.38 (1.15, 1.66)*	**1.45 (0.93, 1.43)**
**Marital Status**:			
**Single**	**241 (32.5)**	**1**	**1**
**Currently-married**	**487 (35.7)**	**1.15 (0.95, 1.39)**	**0.88 (0.72, 1.08)**
**Formerly-married**	**88 (52.1)**	**2.26 (1.61, 3.17)** *	1.55 (1.02, 2.37)*
**Education Status**:			
**Never been to school/Incomplete primary school**	**264(39.2)**	**1**	
**Completed primary school**	**403 (34.4)**	**0.82 (0.67, 1.00)**	
**Completed/In completed secondary school**	**149 (34.5)**	**0.82 (0.64, 1.05)**	
**Having children**:			
**No**	**175 (27.1)**	**1**	**1**
**Yes**	**641 (39.4)**	1.75 (1.43, 2.14)**	1.79 (1.47, 2.16)**
**Anxiety-depression symptoms**:			
**Normal**	**225 (25.9)**	**1**	**1**
**Mild**	**274 (36.1)**	**1.61 (1.31, 2.00)** **	1.63 (1.26, 2.07)**
**Moderate**	**232 (47.3)**	**2.56 (2.03, 3.23)** **	2.57 (2.00, 3.29)**
**Severe**	**85 (53.5)**	**3.33 (2.35, 4.71)** **	3.27 (2.15, 4.96)**
**HIV status**^a^:			
**Negative**	**774 (35.6)**	**1**	
**Positive**	**39 (42.9)**	**1.36 (0.89, 2.08)**	
**Sex work in the last six months**:			
**No**	**625(33.1)**	**1**	**1**
**Yes**	**191 (49.4)**	**1.97 (1.58, 2.46)** **	**1.92 (1.45, 2.53)** *
**Economic deprivation**:			
**Low**	**479 (31.2)**	**1**	**1**
**High**	**337 (45.5)**	**1.85 (1.54, 2.21)** **	**1.61 (1.34, 1.92)** *

^**a**^10 observations missed (AGYW they left field site before HIV testing);

*p<0.05;

**p<0.001

**Fig 2:**
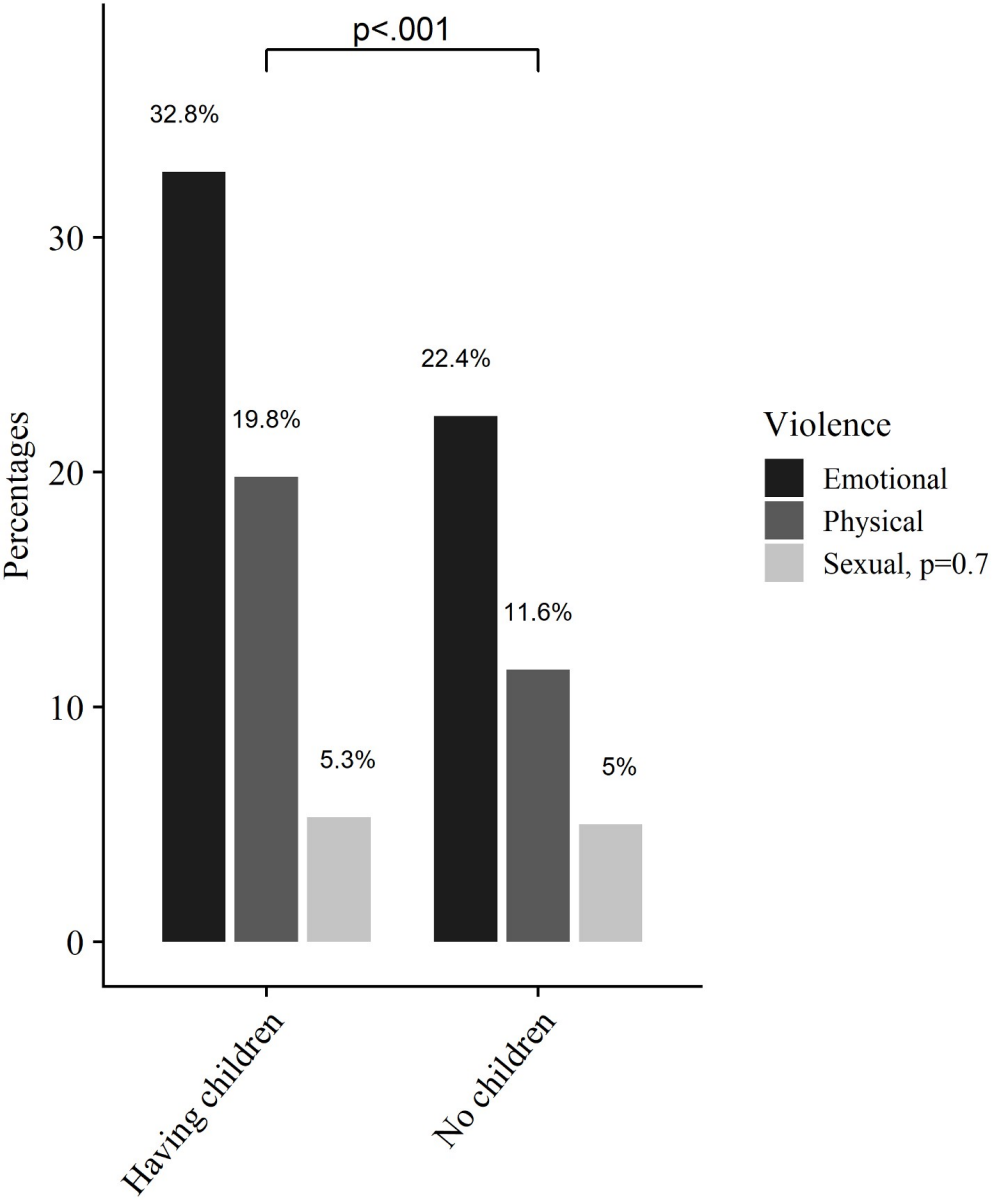
Proportion of violence typologies by parity.

Emotional violence was the highest reported type of violence (e.g. almost 33% experienced emotional violence) among AGYW reporting having children compared to AGYW who did not have children (Fig 2).

### Factors associated with partner violence

In an unadjusted model, age, marital status, having children, anxiety and depression (PHQ-4) symptom, engaging in sex work in the last six months and economic deprivation were significantly associated with reporting experiencing partner violence. After adjusting for other covariates, AGYW who were formerly married were more likely to experience partner violence (AOR = 1.55, 95% CI: 1.02, 2.37) compared to those who were single. AGYW who reported having children were significantly more likely to experience partner violence (AOR = 1.79, 95% CI: 1.47, 2.16) than those who did not have children. Screening positive for symptoms of anxiety and depression was significantly associated with experiencing partner violence. AGYW who screened positive symptoms for severe anxiety and depression were about three times more likely to experience partner violence compared to those who screened negative (AOR = 3.27, 95% CI: 2.15, 4.96).

Similarly, those reporting engaging in sex work in the past six months were more likely to have experienced partner violence (AOR = 1.92, 95% CI: 1.45, 2.53) compared to those who did not engage in sex work. Additionally, those reporting economic deprivation were about two times more likely to have experienced partner violence (AOR = 1.61, 95% CI: 1.34, 1.92) compared to those who did not report deprivation.

Among covariates, education and HIV status were not associated with partner violence. Moreover, after adjusting for other covariates, the association between age and partner violence observed in the univariate analysis was statistically not significant. It was observed that age was strongly associated with having children (χ^2^ = 357.6, P<0.001).

## Discussion

This study provides insight into the extent of partner violence experienced by AGYW. We found that all forms of partner violence (physical, emotional and sexual) among sexually active AGYW were common in the study area. Overall, 35.9% (n = 816) of sexually active AGYW enrolled in the trial reported having experienced partner violence in the last six months. However, this prevalence was 37.5% among ever married. The data is consistent with analyses of partner violence by area-level socioeconomic status and gender-related norms, as reported in surveys about violence against women [38]. The prevalence in this study, however, is lower when compared to the national prevalence among ever-married young women aged 15–24 years, which was 47.7%. However, it should be noted that the national survey asked the experience of violence for the past twelve months [39], while our study inquired experience of violence for the past six months. In addition, this study was conducted among out-of-school girls and young women. Thus, for comparison purposes, it is important that a study adopting similar time frame be conducted among out-of-school girls age 15–23 years.

The prevalence of emotional, physical and sexual violence observed in this population is consistent with previous studies conducted in Tanzania [40, 41], in other sub-Sahara African countries [42] and beyond [43]. Emotional violence was the most common form of violence observed in this study (29.9%), and over a third (33%) of AGYW with children reporting emotional violence, suggesting that this may be normal behaviour in this context. One possible explanation is that couples who enter early into relationships are more likely to face relationship stressors such as early pregnancies and financial difficulties that can lead to partner violence [44]. While more in-depth research to understand the lived experiences on the impact of the different forms of violence, these findings point to the need for comprehensive violence prevention interventions to address partner violence and more so the emotional violence that seems to be a hidden form of violence and thus neglected [45, 46].

Although evidence on the association between partner violence and age and education has been consistently reported elsewhere [2, 4], these characteristics were not significantly associated with partner violence in the current study. An intricate linkage between having children, age, education and partner violence is well established. Women with children can be more tolerant or resilient to violent relationship so that their children can retain a relationship with their father [47–49]. Young women entering in relationships are more likely to be victimised and experience partner violence than mature women [49, 50]. Increased level of education among women can be more empowering as educated women can be more confident and challenge gender stereotypes and less likely to conform to justify IPV and male subjugation [51]. While these linkages have been established for women of the general population, the lack of influence of age and education on partner violence among out-of-school girls involved in this study calls for further in-depth research to provide a nuanced understanding of this finding.

Sexually active AGYW growing up in homes facing economic deprivation (hunger and enrolment in social welfare programs) were more likely to report partner violence experience than those who lived in relatively better off households. This finding is consistent with several other studies which have shown that poverty offers an environment for violence against women to thrive [38, 51, 52]. It is suggested that poor households where men are the only providers, men tend to be perpetrators of violence due to poverty-related stressors that lead to fighting over spending and women’s dependence on men [53]. Equally, where women are not economically disadvantaged, men have been found to resort into violence to reassert control [54, 55]. The pathway through which poverty influence partner violence can be interpreted in the context of power relations in that lack of financial resources and financial dependency on perpetrators increases AGYW’s vulnerability to partner violence. This finding suggests that interventions that focus on reducing AGYWs’ financial dependence and increased autonomy in decision making may reduce the risk of partner violence.

We found that AGYW displaying severe anxiety and depression symptoms were three times more likely to report an experience of partner violence, and such frequency of experience decreased with a reduction in the intensity of symptoms. This finding is supported by studies elsewhere [1, 56], which show that women experiencing partner violence are more likely to have mental health symptoms. Meta-analyses on partner violence and mental health illnesses have confirmed that partner violence increases the risk of mental health problems [17, 57]. However, owing to the nature of the study design, we could not establish the direction of the relationship.

Considering the high prevalence of emotional violence, a life course perspective that emphasises education and early intervention of risk behaviours can have long-term gains in impacting young people’s behaviours that inculcates the culture of mutual respect. This will not only entail engaging young people, rather from parenting to influence individual men and women throughout their lifespan. Evidence has shown that children’s exposure to violence contributes to future partner violence [58, 59]. Although present approaches are essential in addressing partner violence, the extent of their long-term impact in the communities raises concerns.

There is a need to underscore the importance of initiating mental health screening programmes that focus on young women presenting at health facilities for antenatal services. Although this study has limitations by its design, the fact that AGYW presenting symptoms of mental health problems, formerly married and with children experience high partner violence, screening for anxiety and depression can help to design appropriate interventions to counteract immediate and long-term health impact associated with these conditions including partner violence.

These findings show that being formerly married (separated, divorced or widowed) was associated with a higher risk for partner violence than those who were single during the six months before the survey. Although there are limited studies that focus on the history of marriage and partner violence in this context and population, this finding is not new. For example, studies elsewhere have shown that separation from one’s violent partner does not end the violence for women. Instead, married women experience a heightened risk of partner violence after separation [60, 61]. Due to increased risk of violence among widows, some researchers have itemized these women as “widows without rights”–implying the multifaceted forms of violence that these women face in their day-to-day lives [62]. In other low and middle-income countries, widows are susceptible to violence and abuse [63]. As most research focuses on women’s risk for partner violence without disaggregating marital status, this finding underscores the importance of understanding violence as experienced by disparate marital status groups for strategizing effective interventions.

While interventions that focus on males as perpetrators and females as victims of partner violence continue to be popular and necessary, expanding the thinking and further exploring sources of violent relationships is desirable. The influence of being formerly married especially separated couples and parity in the prevalence of partner violence in this study suggest the need for couple counselling and couple communication programmes. Clinical trials have shown that couple counselling function on a systemic level (individual, couple, societal and intergenerational) and is effective when helping couples with dysfunctional relationships that precipitate violence [64, 65]. A systematic review and meta-analysis of 1733 studies have concluded that couple counselling is a viable strategy in addressing violence in select situations such as those emanating from substance abuse [66]. In contexts like Tanzania, where male-partner escort to the health facility for antenatal services is required, it may provide a valuable opportunity for couple counselling on partner violence among other interventions. Additionally, couple counselling through these facilities can have a long-term impact on partner violence and partners’ health in general. In contexts like Tanzania, where men and women condone partner violence, interventions that focus on females or males alone are unlikely to address partner violence adequately.

### Study limitations

Our findings should be interpreted in the context of certain limitations. First, while prevalence and factors associated with partner violence were explored, due to the lack of a qualitative component, causes and contextual factors were not fully explored in this study. Second, data were based on self-report, thus there might be recall bias on some issues, as well as cultural biases in disclosure. Third, the definition of partner violence was within the last 6 months rather than a definition which was inclusive of ever experiencing partner violence. Fourth, the economic abuse and social norms were not measured in this study. Fifth, this paper presents findings from the baseline survey data collected at a one-time point, and thus cannot infer causality.

## Conclusion

This study has found a high prevalence of partner violence among sexually active AGYW aged 15–23 who are out-of-school and that the odds of partner violence were higher in those facing economic deprivation, engaged in sex work, had children, had a history of marriage (currently divorced or separated or widowed), and with anxiety and depression. There is a need for future research to focus on understanding the reasons and dynamics underlying high level of partner violence among AGYW. Unrelenting partner violence and associations with AGYW’s economic deprivation and sex work underscores the need for implementing intervention programs that aim to address AGYWs dependence on partners for survival. Such interventions need to include strategies which will result in economic empowerment of AGYW. Furthermore, it is important that community-based interventions to address social norms and structures perpetuating violence against AGYW are urgently implemented.

## Supporting information

S1 Questions in Kiswahili (DOC)
