# Supplementary material for: Prevalence and correlates of partner violence among adolescent girls and young women: Evidence from baseline data of a cluster randomised trial in Tanzania
Source: PLoS One. 2019 Oct 8;14(10):e0222950. doi: 10.1371/journal.pone.0222950 (PMC6782098; doi:10.1371/journal.pone.0222950)
Supplement: S1 Questions in Kiswahili — (DOC) [file pone.0222950.s001.doc]

**Maswali yanayohusu ukatili wa kijinsia kati ya wapenzi (Swahili version)**

|  | **Questions** | | | | | | | | | |
| --- | --- | --- | --- | --- | --- | --- | --- | --- | --- | --- |
| **Ukatili wa kihisia** | Katika kipindi cha miezi sita iliyopita, je, kuna yeyote kati ya wapenzi wako amewahi:  1. Kukusema au kukufanyia kitu kilichokudhalilisha mbele ya watu?  2. Kutishia kukuumiza au kumuumiza mtu unayemjali?  3. Kukutukana au kukufanya ujisikie vibaya? | | | | | | | | | |
|  |  |  | |  |  |  |  |  | |  |
| **Ukatili wa kimwili** | Katika kipindi cha miezi sita iliyopita, je, kuna yeyote kati ya wapenzi wako amewahi kutumia mikono yake au kitu kingine: | | | | | | | | | |
|  | 1. Kukuumiza mwilini? Hii inahusisha: | | | | |  |  |  |  | |
|  | a. Kukusukuma | | | | | | | | | |
|  | b. Kukutingisha au kukutupia kitu | | | | | | | | | |
|  | c. Kukupiga kibao |  | |  |  |  |  |  |  | |
|  | d. Kukupiga kwa ngumi au kitu kingine kinachoweza kukuumiza | | | | | | |  |  | |
|  | e. Kukupiga teke au kukupiga |  | |  |  |  |  |  |  | |
|  | f. Kukukaba shingoni | | | |  |  |  |  |  | |
|  | g. Kukuunguza kwa makusudi | | | | |  |  |  |  | |
|  | h. Kutishia kukuumiza kwa kisu, bunduki au silaha yeyote | | | | | | |  |  | |
|  |  |  | |  |  |  |  |  |  | |
| **Ukatili wa kingono** | Katika kipindi cha miezi sita iliyopita, je, umewahi kufanyiwa shambulio la kingono na yeyote kati ya wapenzi wako? Ukatili wa kingono ni hali ya: | | | | | | | | | |
|  | 1. Kufanyiwa tendo la ngono kinyume na matakwa yako, hii inajumuisha: | | | | | | | |  | |
|  | a. Ngono kwa njia ya uke au haja kubwa | | | |  |  |  |  |  | |
|  | b. Kuwekwa vidole au vitu vingine  c. Ngono ya mdomoni | |  | |  |  |  |  |  | |
|  |  | |  | |  |  |  |  |  | |
